# Microhardness and flexural strenght of resins for the posterior sector: *in vitro study*

**DOI:** 10.21142/2523-2754-1401-2026-274

**Published:** 2025-12-28

**Authors:** Jerica Alejandra Becerra-Gutiérrez, Ana Isabel López-Flores

**Affiliations:** 1 Stomatology Department, Universidad Científica del Sur. Lima, Perú. jerica.bgutierrez@gmail.com Universidad Científica del Sur Stomatology Department Universidad Científica del Sur Lima Peru jerica.bgutierrez@gmail.com; 2 Oral Rehabilitation Division, Stomatology Department, Universidad Científica del Sur. Lima, Perú. dra.lopz@gmail.com Universidad Científica del Sur Oral Rehabilitation Division Stomatology Department Universidad Científica del Sur Lima Peru dra.lopz@gmail.com

**Keywords:** flexural strength, mechanical properties, surface microhardness, posterior resin composites, resistencia a la flexión, propiedades mecánicas, microdureza superficial, resinas sector posterior

## Abstract

**Introduction.:**

Contemporary resin composites have improved mechanical properties, such as microhardness and flexural strength, to better withstand masticatory loads. Nevertheless, fracture and wear remain common causes of failure in posterior restorations.

**Objective.:**

To compare the surface microhardness and flexural strength of Filtek™ Z350 XT (3M ESPE), Estelite Posterior (Tokuyama), Beautifil II (Shofu), and Opallis (FGM).

**Materials and Methods.:**

In vitro study with four resin composites (n = 10 per group). Disc-shaped specimens (5 mm × 2 mm) were used to evaluate Vickers microhardness (VHN), and bar-shaped specimens (25 mm × 2 mm × 2 mm) to perform a three-point bending test using a universal testing machine.

**Results.:**

For flexural strength, Estelite Posterior showed the highest mean (136.59 ± 9.13 MPa). For surface microhardness, Filtek Z350 XT yielded the highest value (67.99 ± 2.60 VHN). Significant differences were found among groups (p < 0.001), except between Z350 XT and Estelite Posterior for microhardness (p = 0.101) and flexural strength (p = 0.252).

**Conclusion.:**

Filtek Z350 XT and Estelite Posterior exhibited higher surface microhardness and flexural strength compared with Beautifil II and Opallis.

**Clinical Relevance.:**

These results support more informed selection of posterior restorative materials, considering performance and longevity in the oral environment prior to clinical use.

## RESUMEN

**Introducción:**

Las resinas compuestas modernas han mejorado sus propiedades mecánicas, como la microdureza y la resistencia a la flexión frente a las fuerzas masticatorias. La fractura y el desgaste continúan siendo causas comunes de falla en restauraciones posteriores.

**Objetivo:**

Comparar la microdureza superficial y la resistencia a la flexión de las resinas Filtek™ Z350 XT (3M ESPE), Estelite Posterior (Tokuyama), Beautifil II (Shofu) y Opallis (FGM).

**Materiales y métodos:**

Estudio in vitro con cuatro resinas compuestas (n = 10 por grupo). Especímenes en forma de discos de resina (5 mm × 2 mm) se utilizaron para evaluar la microdureza Vickers (VHN) y en forma de barras (25 mm × 2 mm × 2 mm) para evaluar la prueba de flexión a tres puntos en máquina de ensayos universal.

**Resultados:**

En la prueba de resistencia a la flexión, Estelite Posterior mostró el promedio más alto (136,59 ± 9,13 MPa), y Filtek Z350 XT obtuvo el valor más alto en microdureza superficial (67,99 ± 2,60 VHN). Se encontraron diferencias significativas entre los grupos (p < 0,001), excepto entre Z350 XT y Estelite Posterior en microdureza (p = 0,101) y resistencia a la flexión (p = 0,252).

**Conclusión:**

Filtek Z350 XT y Estelite Posterior presentaron mayor microdureza superficial y resistencia a la flexión en comparación con Beautifil II y Opallis.

**Relevancia clínica:**

Los resultados contribuyen a una mejor selección de materiales restauradores, considerando su comportamiento y longevidad en el entorno oral antes del uso clínico.

## INTRODUCTION

Resin composites consist primarily of an organic matrix (dimethacrylate monomers), inorganic filler, and a coupling agent ^1^. Their properties vary with the specific combinations and proportions of monomers, influencing characteristics such as hardness, polymerization shrinkage, and strength ^2^. Recent studies report that newer-generation resin composites perform better under functional stresses than conventional materials. Therefore, restorative materials used in areas subjected to masticatory forces should be evaluated through physical and mechanical testing ^3^.

The main cause of failure in direct posterior restorations is material fracture, which is related to limitations in mechanical properties ^4^. Physical wear also reduces strength, increases surface roughness, and shortens restoration longevity ^5^. Brunthaler et al. published a review of prospective studies identifying the predominant reasons for failure in direct posterior composite restorations, with fracture and secondary caries during the first five years being the leading factors ^6^.

Microhardness is a key mechanical property that measures a material’s resistance to indentation under a localized load. In dentistry, the Vickers method is widely used ^7^^,^^8^. Microhardness can decrease due to wear caused by saliva and masticatory forces, affecting the material’s internal cohesion ^9^. This property is directly related to filler content and polymerization degree; greater filler load and higher conversion generally yield higher surface microhardness ^10^.

Flexural strength is another relevant mechanical property, reflecting the material’s ability to resist deformation and fracture under external forces ^11^. Materials with high flexural strength show less tendency to crack and greater stability against erosive challenges ^12^.

Meenakumari et al. evaluated five posterior composites (SureFil SDR, Clearfil Majesty, EverX, Tetric EvoCeram Bulk Fill, and Filtek Z350) and observed that Filtek Z350 and EverX had the highest microhardness and flexural strength values ^13^. Pala et al. compared nanofilled, nanohybrid, and microhybrid composites, finding that nanofilled materials present higher microhardness, while flexural strength values are similar across groups ^14^.

Finally, recent studies highlight the need to develop restorative materials with physical, chemical, and mechanical properties that approximate those of natural tooth tissues, prioritizing resistance to wear, flexure, and compression ^15^. However, further studies are still needed to evaluate these properties and identify the most suitable materials for posterior restorations. Accordingly, the present study aimed to compare the surface microhardness and flexural strength of four restorative composites indicated for posterior teeth.

## MATERIALS AND METHODS

### Study sample

The sample comprised widely used posterior restorative composites allocated into four groups: one control group with Filtek™ Z350 XT 3M ESPE (G1A) and three experimental groups-Estelite® Posterior Tokuyama (G2A), Beautifil II Shofu (G3A), and Opallis FGM (G4A). Disc-shaped specimens were used for surface microhardness and bar-shaped specimens for flexural strength testing (n = 10 specimens for each brand). Table 1 lists the commercial name, composite type, lot number, expiration date, and chemical composition.

**Table 1:** Composition and characteristics of the restorative materials used

Material	Batch No	Expiration Date	Manufacturer	Type	Composition		
Filtek™ Z350 XT	NF33315	2025/03/20	3M ESPE, St, Paul NM, USA	Nanoparticle	Bis- Gma Bis- Ema USMA y TEGDMA	Nanoparticles of silica 20 nm Zirconia/Silica: 5-2 nm nanocluster: 0,6 to 1,4 µm
Estelite posterior	W1476	2025/07	Tokuyama	Microhybrid	Bis- GMA (TEGDMA) Trietilen glicol dimetacrilato Y Bis-. MPEPP	Particles of silica-zirconio 2 µm 0,1 - 10 µm
Beautifil II	032212	2025/02/28	Shofu, Kyoto. Japan	Giomer Technology	Bis- Gma, TEGDMA, Fluoruroboro-alumicosilicate glass	Particles of 0,8 µm 0,01 - 4 µm
Opallis	251021	2024/10/25	FGM Brasil	Nanohybrid	Bis-GMA, Bis EMA, TEGDMA, UDMA	0,5 µm 2,0 - 3,0 µm

### Sample size

The final sample size was based on pilot data (n = 5 per group) using the formula for comparing two means, with 95% confidence and 80% power. In the pilot, surface microhardness values were 48.2 ± 2.54 VHN for Opallis and 68.32 ± 0.43 VHN for Filtek Z350 XT. Flexural strength values were 77.33 ± 0.16 MPa for Opallis and 133.54 ± 0.29 MPa for Filtek Z350 XT. This yielded 5 discs and 5 bars per resin group. Pilot specimens were included in the definitive analysis.

### Preparation of discs

**Figure 1:**
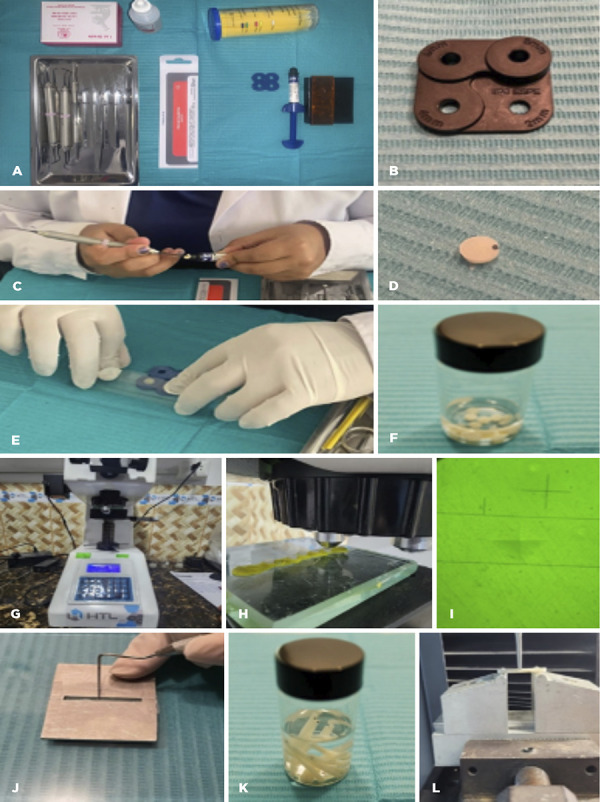
Experimental workflow: 3M mold for disc fabrication (A); composite placement with Hu-Friedy instrument (B); surface parallelization with matrix strip and glass slide (C); finished/polished disc (D); disc storage (E); Vickers microhardness testing (F); indent placement (G); indentation (H); mold for bar fabrication (I); bar fabrication (J); bar storage (K); three-point bending on universal testing machine (L).

Discs were fabricated using a 3M ESPE mold (Figure 1A) with 5 mm diameter and 2 mm thickness, following ISO 4049 standardization ^16^. A thin film of liquid vaseline was applied to the mold cavity with a microbrush. A glass slide was placed at the base to ensure parallel surfaces, and the composite was inserted as a single increment using a Teflon-coated composite instrument (Hu-Friedy) (Figure 1B). Prior to light curing, a polyester matrix strip and a glass slide were placed on the surface and gently compressed with a finger (Figure 1C). Photoactivation was performed with an ELIPAR LED curing light (3M ESPE, St. Paul, USA) for 20 s at 1200 mW/cm², followed by finishing/polishing with Sof-Lex discs (3M ESPE, St. Paul, USA). Specimens were stored in physiological saline solution beginning 24 h after fabrication (Figure 1E).

### Microhardness testing

A Vickers microhardness tester (H-V 1000) (Figure 1F) was used. Vickers hardness was calculated as HV = 1.8544 × F / d². A 200 g load was applied for 10 s (Figure 1G) per indentation. Three indentations (Figure 1H) were made on each disc surface, and their mean was taken as the surface microhardness for that specimen.

### Preparation of bars

Bars were fabricated in a stainless-steel mold (25 mm × 2 mm × 2 mm) according to ISO 4049, using two increments (Figure 1G). A thin layer of liquid vaseline was applied to the mold with a microbrush to prevent adhesion. Composite was placed with a Teflon-coated instrument (Hu-Friedy). Before curing, a glass slide was positioned to parallelize the surfaces. The ELIPAR LED curing light (3M ESPE St. Paul, EE. UU) was applied in the central, right, and left regions for 20 s each, at 1200 mW/cm² ^17^, with output verified every five specimens using a One Cure O-Star (Woodpecker) radiometer to maintain a consistent irradiance. Specimens were removed and finished/polished with Sof-Lex discs. Bars were stored in physiological saline solution starting 24 h after fabrication (Figure 1J).

### Flexural strength testing

Flexural strength was determined on a CMT5L universal testing machine (Figure 1K) at a crosshead speed of 0.5 mm/min until fracture. Bars were placed on a metal fixture with a 20-mm support span, and load was applied at the midpoint using the three-point bending method.

### Data analysis

Statistical analyses were performed in SigmaPlot version 12 at a 95% confidence level. Descriptive statistics for surface microhardness and flexural strength included mean, standard deviation, variance, minimum, and maximum. Data normality was assessed with the Shapiro-Wilk test. One-way ANOVA and Tukey’s HSD multiple comparisons were used for inferential analysis.

## RESULTS

For flexural strength, Estelite Posterior (microhybrid) showed the highest mean (136.59 ± 9.13 MPa), followed by Z350 XT (129.67 ± 5.87 MPa), Beautifil II (106.08 ± 6.05 MPa), and Opallis (89.22 ± 10.87 MPa). For surface microhardness, Filtek Z350 XT (nanofilled) exhibited the highest mean (67.99 ± 2.60 VHN), followed by Estelite Posterior (64.99 ± 1.60 VHN), Beautifil II (57.83 ± 2.07 VHN), and Opallis (44.55 ± 4.28 VHN).

When comparing surface microhardness among groups, statistically significant differences were found between Z350 XT and Beautifil II (p < 0.001), Z350 XT and Opallis (p < 0.001), Estelite Posterior and Beautifil II (p < 0.001), Estelite Posterior and Opallis (p < 0.001), and Beautifil II and Opallis (p < 0.001), except between Z350 XT and Estelite Posterior (p = 0.101) (Table 2).

**Table 2:** Surface microhardness (VHN) of four posterior restorative resin composites

Material	n	Mean	SD	Min	Max	S2	P1	P2
1. Z350 XT	10	67,99	2,60	63,2	72	0,39	<0,001	0,101 between 1 y 2
2. Estelite posterior	10	64,99	1,60	62,5	67,5	0,90		<0,001 between 1 y 3
								<0,001 between 1 y 4
3. Beautifill II	10	57,83	2,07	55,3	61,5	0,52		<0,001 between 2 y 3
								<0,001 between 2 y 4
4. Opallis	10	44,55	4,28	37,1	51,1	0,97		<0,001 between 3 y 4

P1: One-way ANOVA; P2: Tukey’s HSD.

For flexural strength, significant differences were observed between Z350 XT and Beautifil II (p < 0.001), Z350 XT and Opallis (p < 0.001), Estelite Posterior and Beautifil II (p < 0.001), Estelite Posterior and Opallis (p < 0.001), and Beautifil II and Opallis (p < 0.001), except between Z350 XT and Estelite Posterior (p = 0.252) (Table 3).

**Table 3:** Flexural strength (MPa) of four posterior restorative resin composites

Material	n	Mean	SD	Min	Max	S2	P1	P2
1. Z350 XT	10	129,67	5,87	121,56	137,84	0,52	<0,001	0,252 between 1 y 2
2. Estelite posterior	10	136,59	9,13	124,27	157,77	0,18		<0,001 between 1 y 3
								<0,001 between 1 y 4
3. Beautifill II	10	106,08	6,05	98,73	115,73	0,29		<0,001 between 2 y 3
4. Opallis	10	89,22	10,87	77,28	113,36	0,14		<0,001 between 2 y 4
								<0,001 between 3 y 4

P1: One-way ANOVA; P2: Tukey’s HSD.

## DISCUSSION

Advances in the chemical formulation of resin composites, particularly in the inorganic filler phase to improve physical and mechanical behavior, have broadened their clinical indications in both anterior and posterior teeth. Given the constant introduction of new brands and formulations, continued evaluation of these properties remains necessary. Accordingly, this study assessed the surface microhardness and flexural strength of four posterior restorative composites: Filtek™ Z350 XT (3M ESPE), Estelite® Posterior (Tokuyama), Beautifil II (Shofu), and Opallis (FGM).

The present results showed significant differences in surface microhardness among all groups except between Estelite Posterior and Filtek Z350 XT, which recorded the highest values. In nanofilled composites such as Filtek Z350 XT, the incorporation of zirconia particles may contribute to the higher microhardness values ^18^^-^^20^. These findings agree with Rizzante et al. ^21^, who compared surface microhardness and depth of cure of conventional composites and bulk-fill materials and reported that conventional Z350 XT exhibited the highest microhardness, likely related to its nanoparticle system, which, per the manufacturer, enhances composite strength due to improved particle distribution and interaction. For Estelite Posterior (a supra-nanohybrid), Burcu et al. ^18^ found that its surface microhardness was significantly higher than that of the comparator, regardless of curing mode-an effect attributed to higher filler loading; inorganic filler content correlates positively with microhardness.

Similarly, flexural strength results showed significant differences across groups except between Estelite Posterior and Filtek Z350 XT, which achieved the highest values. Botre et al. ^19^, evaluating physical-mechanical properties after additional polymerization, reported higher flexural strength for Filtek Z350 XT compared with other materials. Burcu et al. ^18^ also noted that flexural properties can be influenced by filler size; in their study, the nanohybrid exhibited significantly lower flexural strength than Estelite Posterior, a finding attributed to lower silicate glass filler content in the nanohybrid.

Fillers are responsible for reinforcing the resin matrix, contributing to translucency, and reducing polymerization shrinkage. Higher filler loading facilitates clinical handling of composites. In general, nanofilled composites present favorable mechanical properties ^20^.

Significant differences were observed for both microhardness and flexural strength in Opallis and Beautifil II, which showed the lowest results. This aligns with reports indicating that microfilled composites exhibit significantly lower mechanical properties than nanofilled and universal hybrid composites ^21^.

In the study by Hashemikamanga et al. ^22^, which compared the surface microhardness of a self-adhesive composite with that of other conventional composites, results suggested that surface microhardness depends largely on the type of material and is affected by the quality of the matrix-filler system; as the filler volume fraction increases, so do surface microhardness and flexural strength. They also noted that filler type may explain higher microhardness when the inorganic phase includes crystalline silica and zirconia-consistent with our findings, in which composites containing silica and zirconia particles in the inorganic filler achieved higher surface microhardness and flexural strength.

Composite failures are primarily attributed to the behavior of different resin matrices and to filler type and percentage. It is important to consider each composite according to its specific clinical indication ^23^. In this context, the present results show that the mechanical properties evaluated were significantly higher for Filtek Z350 XT and Estelite Posterior, suggesting better clinical performance for posterior restorations.

### Study limitations

As an in vitro investigation, these results are not directly generalizable to clinical conditions, which is the main limitation. Nevertheless, standardized protocols were followed, and the mechanical property values obtained are comparable to those reported in the literature. Thus, the findings may help inform material selection according to the clinical demands of posterior restorations. Further research assessing additional physical and mechanical properties-across these and other commercially available composites-is warranted to complement the present results and to strengthen translation to clinical practice.

## CONCLUSIONS

Surface microhardness and flexural strength of Filtek Z350 XT and Estelite Posterior were significantly higher than those of Beautifil II and Opallis.

## Financing:

Self-financed
